# Whole-genome sequencing to investigate a non-clonal melioidosis cluster on a remote Australian island

**DOI:** 10.1099/mgen.0.000117

**Published:** 2017-06-13

**Authors:** Derek S. Sarovich, Stephanie N. J. Chapple, Erin P. Price, Mark Mayo, Matthew T. G. Holden, Sharon J. Peacock, Bart J. Currie

**Affiliations:** ^1^​ Global and Tropical Health Division, Menzies School of Health Research, Darwin, Australia; ^2^​ Centre for Animal Health Innovation, University of the Sunshine Coast, Sippy Downs, Australia; ^3^​ Melbourne Medical School, University of Melbourne, Melbourne, Australia; ^4^​ School of Medicine, Medical and Biological Sciences, University of St Andrews, St Andrews, UK; ^5^​ Wellcome Trust Sanger Institute, Cambridge, UK; ^6^​ Department of Medicine, University of Cambridge, Cambridge, UK; ^7^​ Department of Infectious Diseases and Northern Territory Medical Program, Royal Darwin Hospital, Darwin, Australia

**Keywords:** melioidosis, source tracing, *Burkholderia pseudomallei*, recombination, population genetics, outbreak

## Abstract

Melioidosis is a tropical disease caused by the bacterium *Burkholderia pseudomallei*. Outbreaks are uncommon and can generally be attributed to a single point source and strain. We used whole-genome sequencing to analyse *B. pseudomallei* isolates collected from an historical 2-year long case cluster that occurred in a remote northern Australian indigenous island community, where infections were previously linked to a contaminated communal water supply. We analysed the genome-wide relatedness of the two most common multilocus sequence types (STs) involved in the outbreak, STs 125 and 126. This analysis showed that although these STs were closely related on a whole-genome level, they demonstrated evidence of multiple recombination events that were unlikely to have occurred over the timeframe of the outbreak. Based on epidemiological and genetic data, we also identified two additional patients not previously associated with this outbreak. Our results confirm the previous hypothesis that a single unchlorinated water source harbouring multiple *B. pseudomallei* strains was linked to the outbreak, and that increased melioidosis risk in this community was associated with *Piper methysticum* root (kava) consumption.

## Abbreviations

HPD, highest posterior density; indel, small insertion/deletion; MLST, multilocus sequence typing; PFGE, pulsed-field gel electrophoresis; SLV, single-locus variant; SNP, single-nucleotide polymorphism; SRA, Sequence Read Archive; ST, sequence type; TMRCA, time since the most recent common ancestor; WGS, whole-genome sequencing.

## Data Summary

Whole genome sequencing data have been deposited into the Sequence Read Archive or the European Nucleotide Archive:

- ERR311046, http://www.ebi.ac.uk/ena/data/view/ERR311046
- SRR3525374, https://trace.ncbi.nlm.nih.gov/Traces/sra/?run=SRR3525374
- SRR3525385, https://trace.ncbi.nlm.nih.gov/Traces/sra/?run=SRR3525385
- SRR3525386, https://trace.ncbi.nlm.nih.gov/Traces/sra/?run=SRR3525386
- ERR311049, http://www.ebi.ac.uk/ena/data/view/ERR311049
- ERR311050, http://www.ebi.ac.uk/ena/data/view/ERR311050
- SRR3525387, https://trace.ncbi.nlm.nih.gov/Traces/sra/?run=SRR3525387
- SRR3525399, https://trace.ncbi.nlm.nih.gov/Traces/sra/?run=SRR3525399
- SRR3525400, https://trace.ncbi.nlm.nih.gov/Traces/sra/?run=SRR3525400
- SRR3525401, https://trace.ncbi.nlm.nih.gov/Traces/sra/?run=SRR3525401
- ERR311053, http://www.ebi.ac.uk/ena/data/view/ERR311053
- ERR311054, http://www.ebi.ac.uk/ena/data/view/ERR311054
- SRR5260553, https://trace.ncbi.nlm.nih.gov/Traces/sra/?run=SRR5260553

## Impact Statement

The tropical disease melioidosis is caused by the soil-dwelling bacterium *Burkholderia pseudomallei. B. pseudomallei* rarely transmits person-to-person, with infections almost exclusively acquired by contact with contaminated water or soil. Due in part to this restricted transmission, melioidosis outbreaks are uncommon, and are typically caused by a single bacterial strain that can be linked to a contaminated point source shared by multiple individuals. In this study, we revisited a historical melioidosis outbreak on a remote Australian island by applying whole-genome sequencing (WGS) to better understand the aetiology of this outbreak. Our analysis shed new light on the outbreak, clarifying transmission patterns and confirming that the outbreak was caused by multiple *B. pseudomallei* strains. In addition, we identified cases not previously known to be associated with this outbreak. Our study provides another example of the utility of WGS for understanding the origin and risk factors associated with bacterial outbreaks.

## Introduction

*Burkholderia pseudomallei* is a Gram-negative soil-borne bacterium and the aetiological agent of melioidosis, a disease endemic in northern Australia and South-East Asia that is becoming increasingly recognized elsewhere [1, 2]. In some endemic regions, *B. pseudomallei* is a major cause of community-acquired pneumonia and, if left untreated, can cause rapid death from septic shock [3]. Person-to-person transmission is extremely rare, with case clusters typically arising following contamination of a common environmental point source. Epidemiological tracking of melioidosis case clusters has traditionally relied on several different genotyping methods, including multilocus sequencing typing (MLST), multilocus variable-number tandem repeat analysis [4, 5] or pulsed-field gel electrophoresis (PFGE) [6–8]. However, these methods are often insufficient for detecting fine-scale genetic variation and can be subject to homoplasy, thereby potentially obscuring the source and time course of suspected outbreaks. These genotyping methods are now being superseded by more modern methods such as whole-genome sequencing (WGS), which provides base-pair level resolution of strain variation across the entire genome [9, 10].

Over a 28 month period in the mid-1990s, a case cluster of nine melioidosis cases with four deaths occurred in a remote island indigenous community in the ‘Top End’ of the Northern Territory, Australia [6]. Following the outbreak, extensive environmental sampling of both soil and environmental water sources was undertaken in an effort to identify the point source of the outbreak [6]. A total of 6 of 87 soil samples and 1 of 35 water samples were positive for *B. pseudomallei*; however, only a single isolate from the community’s unchlorinated water supply storage tank matched the clinical case isolates using PFGE [6]. MLST on a subset of these outbreak isolates revealed two different sequence types (STs): ST-125 and its single-locus variant (SLV), ST-126 [11].

In the current study, we built upon this previous work [6, 11] by characterizing this historical outbreak using both MLST and WGS. Our genomic analysis of these strains included additional comparisons with our larger clinical dataset from the Darwin Prospective Melioidosis Study [12], which at the time of writing is in its 27th year.

## Methods

### Bacterial growth, extraction and DNA sequencing

DNA was extracted from purified *B. pseudomallei* colonies as previously described [11]. WGS was performed on the Illumina HiSeq2000 or HiSeq2500 platforms (Illumina). Read data were deposited into the Sequence Read Archive (SRA) database or European Nucleotide Archive (ENA; Table 1).

**Table 1.** Patients included in the case cluster and associated *B. pseudomallei* strains

Date of isolation	Patient ID/source	Died	Isolate	Location*	ST	ERA/SRA accession no.
Mar 1994	P97†	No	MSHR0287	A	125	ERR311046
Mar 1994	P96†,‡	No	MSHR0286	B	125	SRR3525374
Nov 1994	P105†	Yes	MSHR0344, MSHR0345	A	125§	SRR3525385, SRR3525386
Jan 1995	P110	Yes	MSHR0356	A	149	ERR311049
Feb 1995	P114†	No	MSHR0362	A	141	na
Mar 1996	P137	Yes	MSHR0445A	A	126§	ERR311050
Apr 1996	P142†	Yes	MSHR0433	A	126§	SRR3525387
Apr 1996	P143†	No	MSHR0435	A	126§	SRR3525399
Apr 1996	P144†,‡	No	MSHR0436	C	125	SRR3525400
Apr 1996	P157†,||	No	MSHR0462	B	126	SRR3525401
June 1996	P150†	No	MSHR0449	A	126§	ERR311053
Mar 1997	Water tank	na	MSHR0491	A	126§	ERR311054
Mar 1997	Soil	na	MSHR0502	A	114	na
Mar 1997	Soil	na	MSHR0503	A	149	SRR5260553
Mar 1997	Soil	na	MSHR0504	A	268	na
Mar 1997	Soil	na	MSHR0505	A	682	na
Mar 1997	Soil	na	MSHR0507	A	719	na
Mar 1997	Soil	na	MSHR0508	A	281	na

na, Not applicable.

*A, Remote island community with contaminated water supply; B and C, other remote communities ~100 km from the outbreak community that were not previously associated with the outbreak.

†Patients reported kava use.

‡Patients were not initially recognized as part of the case cluster. These cases were included in the analysis due to the infecting strains being genetically linked to other case cluster strains, and the patients being epidemiologically linked by kava use and geographical proximity.

§STs previously reported for this patient or environmental sample [11].

||Patient was initially considered part of the remote island community case cluster, but was travelling on the mainland at the time of diagnosis.

### Comparative genomic analysis

The high-quality, finished genome of the water supply storage tank isolate MSHR0491 [13] was used as the reference (GenBank accession numbers CP009485.1 and CP009484.1; SRA accession number ERR311054). Comparative genomic analysis was performed using bwa v0.6.2 [14] for read alignment, SAMTools v1.2 [14] and Picard (https://broadinstitute.github.io/picard/) for alignment processing, gatk 3.2.2 [15] for single-nucleotide polymorphism (SNP), small insertion/deletion (indel) identification and comparative analysis of variants, and SnpEff v4.1 [16] for variant annotation. These programs were executed as part of the SPANDx pipeline v3.1.2 [17], which wraps these tools for ease-of-use and reproducibility. Regions of recombination were identified using Gubbins v1.4.1 [18] and ClonalFrameML [19]. Phylogenetic reconstruction was performed using maximum parsimony in paup v4.0a142 [20] or maximum likelihood in RAxML v8.1.17 [21], with resultant trees visualized and manipulated using FigTree v1.4.0 (http://tree.bio.ed.ac.uk/software/figtree/).

Genomes were assembled to improved high-quality draft standard [22] using the mgap pipeline (https://github.com/dsarov/MGAP-Microbial-Genome-Assembler-Pipeline), which consists of Trimmomatic [23] and Velvet [24], with parameters optimized using VelvetOptimiser (https://github.com/tseemann/VelvetOptimiser), and subsequent draft improvement with sspace [25], Gapfiller [26], image [27] and icorn2 [28]. MLST profiles were extracted from whole-genome assemblies using BIGSdb [29], which is available on the *B. pseudomallei* PubMLST website (https://pubmlst.org/bpseudomallei/).

## Results and Discussion

Between March 1994 and June 1996, an unusual case cluster of nine melioidosis cases occurred in a remote island indigenous community in the Northern Territory, Australia [6]. Shortly following the outbreak, extensive environmental sampling was undertaken in an attempt to identify the source of infection [6]. PFGE was performed on the clinical isolates from the outbreak, with the resulting profiles showing that the majority of strains (7/9) were identical. The PFGE profile of an environmental isolate from the community water supply matched the clinical isolates, leading to the hypothesis that the outbreak was caused by contamination of the unchlorinated water supply [6]. A subsequent study using MLST on a subset of these isolates (*n*=6) showed that, despite these strains being identical by PFGE, they actually consisted of two different STs, ST-125 and ST-126 [11].

In the current study, we performed MLST on all available isolates (*n*=19) associated with the outbreak. These isolates comprised 12 clinical strains [5 with prior MLST data [11] and 1 environmental isolate from the community water supply (MSHR0491; also previously typed by MLST [11])] and 6 additional environmental isolates derived from soil sampling conducted shortly following the outbreak. MLST showed that the clinical isolates belonged to one of four STs: STs 125 (*n*=4), 126 (*n*=5), 141 (*n*=1) or 149 (*n*=1) (Table 1). ST-125 and ST-126 are SLVs according to MLST, being identical at all but the *ndh* locus, where they differ by two SNPs. ST-141 and ST-149 appear unrelated to each other, with only 1/7 loci in common, and unrelated to ST-125 and ST-126, with only the *ndh* locus shared between ST-125 and ST-149. Two identical MLST profiles were identified among the environmental and clinical isolates. As reported previously [11], the community water supply isolate MSHR0491 was ST-126, as were five of the clinical strains, supporting the initial conclusion reached with just PFGE data. The second match that was identified occurred between one of the soil isolates (MSHR0503; ST-149), and one of the clinical isolates (P110, MSHR0356; ST-149). The remaining five soil isolates had unrelated MLST profiles compared with the clinical strains. The two other STs observed in clinical isolates from the outbreak, ST-125 and ST-141, could not be attributed to an environmental source (Table 1).

To better understand the aetiology of this melioidosis case cluster, we performed WGS of all ST-125 (*n*=5), ST-126 (*n*=6) and ST-149 (*n*=2) strains. Nine isolates were from eight case cluster patients (two isolates were from one patient), two were environmental isolates that matched with MLST to clinical cases and the remaining two were from melioidosis patients living in two separate communities, each located ~100 km from the remote island case cluster, who had not originally been linked to the case cluster but were identified as also being infected with ST-125 strains through the course of the Darwin Prospective Melioidosis Study [12] (Table 1).

**Fig. 1.**
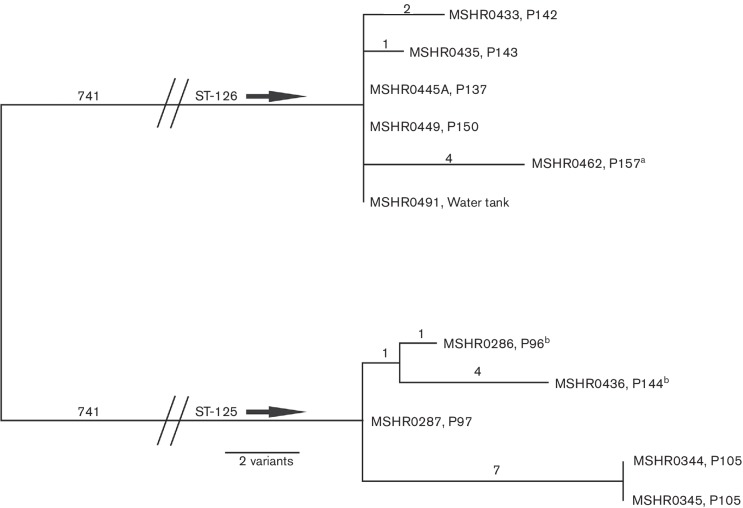
Mid-point rooted maximum-parsimony phylogenetic reconstruction of ST−125 and ST-126 isolates from a remote indigenous island community case cluster using a combination of SNPs and indels. Note that despite including both indel and SNP variation, within-ST diversity remains small. Branches separating ST-125 and ST-126 have been collapsed to enable better visualization of within-ST diversity. ST-125 and ST-126 isolates are separated by 1328 SNPs or 1482 SNP-indel variants. a, P157 was originally included as part of the case cluster, but was travelling on the mainland at the time of diagnosis; b, P96 and P144 were not originally recognized as part of the case cluster, but were subsequently included due to genetic and epidemiological links including kava use.

WGS analysis of ST-125 and ST-126 isolates showed that there was little within-ST variation on a whole-genome scale. For the ST-126 isolates, one SNP differentiated MSHR0435 from the other ST-126 isolates, which were otherwise indistinguishable (data not shown). Using a combination of SNPs and indels to increase resolution [10, 30], three (50 %) of the ST-126 isolates could be differentiated (Fig. 1). Within-ST diversity for ST-125 was similarly minimal; the entire ST-125 population differed by only 2 SNPs and 11 indels (Fig. 1). Of note, the two isolates from P105 were identical, yet differed from other ST-125 isolates by seven variants. These two isolates also lacked a~24 kb region of chromosome II (position ~821 000 to ~845 000 bp) due to a large deletion that was not detected with PFGE (Fig. S1, available in the online Supplementary Material), and which was not observed in any of the other isolates (data not shown). It is possible that the isolates from P105 had undergone within-host evolution [31, 32] or, alternatively, that there was a mixed population of ST-125 isolates in the communal water supply [33]; due to limited sampling performed at the time of collection, the precise scenario cannot be ascertained.

Despite PFGE being unable to discriminate between ST-125 and ST-126 [6] (Fig. S1), these STs differ by at least 1328 SNPs and 154 indels on the whole-genome level (Fig. 1). *B. pseudomallei* has a highly syntenic genome, even among distantly related strains [34]; thus, it is not altogether surprising that PFGE did not detect differences between these STs. Interestingly, the genetic distance observed between these STs was less than that observed within some STs, suggesting relatively recent divergence. For example, whole-genome analysis of 36 ST-109 isolates from Darwin, Northern Territory, Australia, showed high SNP diversity (~9500 SNPs), likely due to the contribution of both recombination and mutation over evolutionary time [35]. Thus, ST-125 and ST-126 are clearly genetically related, which concurs with their MLST status as SLVs.

*B. pseudomallei* is a highly recombinogenic species and substantial genetic differentiation can arise due to recombination events [33, 36], which can confound evolutionary signal. To investigate this possibility in the current dataset, the program Gubbins [18] was used to identify putative recombination events among the ST-125 and ST-126 isolates. Clustering of recombinogenic SNPs in genome ‘hotspots’ occurred at 36 discrete recombination blocks totalling ~339 kb (Figs S2 and S3, Table S1). A similar observation was made using ClonalFrameML [19], with 39 discrete recombination blocks totalling ~333 kb being identified (data not shown). Following removal of recombinogenic regions, 73 SNPs (~5 % total) still separated the ST-125 and ST-126 strains. To determine the time since the most recent common ancestor (TMRCA) of the ST-125 and ST-126 strains, we applied a substitution rate calculated previously for Asian populations of *B. pseudomallei* [37]. Based on a substitution rate of 1.03×10^−6^ per site per year [95 % highest posterior density (HPD)=6.38×10^−7^ to 1.32×10^−6^] [37] and a genome size of 7.0 Mbp (i.e. the size of the ST-125/ST-126 *B. pseudomallei* genome with the recombined regions removed), the TMRCA is 5 years (95 % HPD=8 to 4 years). Previously described substitution rates for *Burkholderia dolosa* populations evolving within human hosts have been estimated to be somewhat slower at 3×10^−7^ substitutions per site per year [38]. Calculation of TMRCA using this slower rate would place divergence at 17 years. Regardless of the chosen substitution rate, these results suggest that divergence between ST-125 and ST-126 did not occur during the timeframe of the case cluster.

The ST-125 isolates obtained from the two patients not originally identified as part of the case cluster (MSHR0286 and MSHR0436) were remarkably similar to other ST-125 isolates on the whole-genome level, differing by only two and five variants, respectively (Fig. 1). Despite living in two separate communities, these cases had family ties to the remote island community and both were kava drinkers, as were seven of the nine island community patients (Table 1). Kava is prepared by infusing cold or warm water with the powdered root of the south-western Pacific Island native plant *Piper methysticum*, and its use is a known risk factor for melioidosis [39]. The strong association between the outbreak and kava consumption was noticed in the original study in 2001 [6], even before the identification of the additional kava-associated cases. The strong link between reported kava use and melioidosis cases in this outbreak further strengthens the hypothesis that the contaminated, unchlorinated community water supply was used for kava preparation. However, three samples of prepared kava obtained from this community at the time of the investigation failed to yield *B. pseudomallei* [6]. It, therefore, cannot be established whether the kava preparation, or the powdered kava itself, was the source of infection, or whether kava users are generally at a greater risk of contracting melioidosis due to, for example, immunosuppression. A better understanding of the effects of kava use on the immune system would help to resolve the basis for the increased melioidosis risk in those who consume kava.

MLST of the *B. pseudomallei* isolates derived from the soil identified a putative link between the environmental soil isolate (MSHR0503) and the clinical isolate from P110 (MSHR0356), both of which were identified as ST-149. However, WGS analysis showed that these strains differed by 57 variants (29 SNPs and 28 indels), indicating that the soil-borne ST-149 population is diverse and that the infecting strain was not sampled at the time. Interestingly, this patient was one of only two involved in the outbreak who did not have a history of recent kava consumption, supporting that this patient likely did not contract melioidosis from the water supply, but was an isolated, endemic case contracted from contaminated soil that occurred concurrently with the outbreak. The only other patient to not list kava as a risk factor presented with septic shock and died shortly after hospital admission, with history of kava use not ascertained.

Due to the historic nature of the outbreak, the soil and water samples collected for the investigation were not preserved for later analysis, so could not be revisited for this study. Fortunately, sampling efforts performed shortly following the outbreak were relatively comprehensive, and involved collection and testing of 87 soil samples and 35 water samples. Of these, only seven were positive for *B. pseudomallei*, with one water sample and six soil samples yielding viable cultures [6]. Both ST-126 and ST-149 isolates were identified in the environmental samples, but the precise origin of the ST-125 strains that infected four of the nine outbreak cases was not determined. Techniques at the time of sampling involved purification of *B. pseudomallei* cultures through a single-colony bottleneck prior to culture storage and genotyping, a strategy that has potentially masked the presence of a genetically heterogeneous population. The clinical history of this outbreak points to an environmental reservoir for ST-125, which may have existed as a mixed population within the community water supply and was simply not identified due to sampling methodology. Subsequent to the recognition of contamination of the water supply with *B. pseudomallei*, the community water supply was remediated and chlorinated. While sporadic endemic cases of melioidosis are still documented from that region, there have been no further cases with ST-125 or ST-126.

In conclusion, we report, to the best of our knowledge, the first documentation of a heterogeneous melioidosis case cluster characterized using WGS. This high-resolution method enabled the investigation of genome-wide strain relatedness and the contribution of recombination in epidemiologically linked SLVs, and in combination with epidemiological evidence, identified kava use as either the probable point source of infection or an associated risk factor. Given the genetic diversity of infecting isolates, this case cluster was likely caused by a polyclonal *B. pseudomallei* population that had contaminated the unchlorinated community water supply, which was then unwittingly ingested by multiple community members over a 2 year period. Concurrent with this outbreak, two sporadic cases occurred in this remote community, most likely due to exposure to contaminated soil. Our study provides an important demonstration of the additional epidemiological and evolutionary insights gained from WGS compared with traditional genotyping methodologies.

## Data bibliography

1. ENA, accession no. ERR311046, http://www.ebi.ac.uk/ena/data/view/ERR311046.
2. NCBI SRA, accession no. SRR3525374, https://trace.ncbi.nlm.nih.gov/Traces/sra/?run=SRR3525374.
3. NCBI SRA, accession no. SRR3525385, https://trace.ncbi.nlm.nih.gov/Traces/sra/?run=SRR3525385.
4. NCBI SRA, accession no. SRR3525386 https://trace.ncbi.nlm.nih.gov/Traces/sra/?run=SRR3525386.
5. ENA, accession no. ERR311049, http://www.ebi.ac.uk/ena/data/view/ERR311049.
6. ENA, accession no. ERR311050, http://www.ebi.ac.uk/ena/data/view/ERR311050.
7. NCBI SRA, accession no. SRR3525387, https://trace.ncbi.nlm.nih.gov/Traces/sra/?run=SRR3525387.
8. NCBI SRA, accession no. SRR3525399, https://trace.ncbi.nlm.nih.gov/Traces/sra/?run=SRR3525399.
9. NCBI SRA, accession no. SRR3525400, https://trace.ncbi.nlm.nih.gov/Traces/sra/?run=SRR3525400.
10. NCBI SRA, accession no. SRR3525401, https://trace.ncbi.nlm.nih.gov/Traces/sra/?run=SRR3525401.
11. ENA, accession no. ERR311053, http://www.ebi.ac.uk/ena/data/view/ERR311053.
12. ENA, accession no. ERR311054, http://www.ebi.ac.uk/ena/data/view/ERR311054.
13. NCBI SRA, accession no. SRR5260553, https://trace.ncbi.nlm.nih.gov/Traces/sra/?run=SRR5260553.

## Supplementary Data

877 Supplementary File 1
