# Supplementary material for: Whole-genome sequencing to investigate a non-clonal melioidosis cluster on a remote Australian island
Source: Microb Genom. 2017 Jun 13;3(8):e000117. doi: 10.1099/mgen.0.000117 (PMC5610713; doi:10.1099/mgen.0.000117)
Supplement: Supplementary File 1 [file mgen-3-117-s001.pdf]

## Figure Legends

**Fig S1. Pulsed-field gel electrophoresis analysis using *SpeI* of environmental and clinical *Burkholderia pseudomallei* isolates involved in a case cluster occurring in a remote island community.** Note that both ST-125 and ST-126 isolates appear identical using this typing method and led to the original conclusion of a clonal outbreak (Currie *et al.,* 2001). A similar figure was published previously in the original study (Currie *et al*., 2001); however, strain names, environmental strains and some clinical strains were omitted from the original figure but have been included in this figure for clarity.

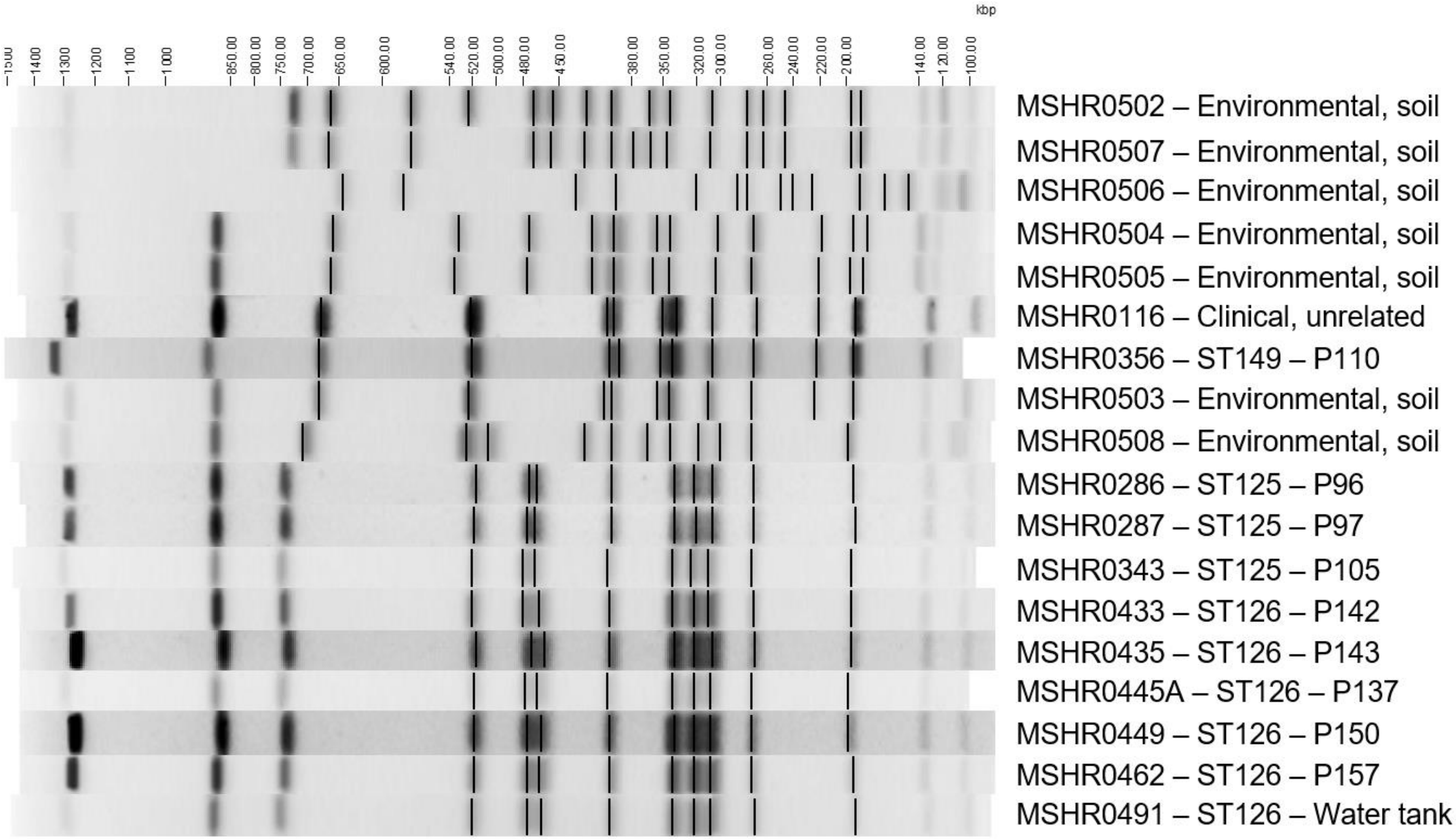
kbp
1400
1300
1200
1100
1000
850.00
800.00
750.00
700.00
650.00
600.00
540.00
520.00
500.00
480.00
450.00
380.00
350.00
320.00
300.00
260.00
240.00
220.00
200.00
140.00
120.00
100.00
MSHR0502 – Environmental, soil
MSHR0507 – Environmental, soil
MSHR0506 – Environmental, soil
MSHR0504 – Environmental, soil
MSHR0505 – Environmental, soil
MSHR0116 – Clinical, unrelated
MSHR0356 – ST149 – P110
MSHR0503 – Environmental, soil
MSHR0508 – Environmental, soil
MSHR0286 – ST125 – P96
MSHR0287 – ST125 – P97
MSHR0343 – ST125 – P105
MSHR0433 – ST126 – P142
MSHR0435 – ST126 – P143
MSHR0445A – ST126 – P137
MSHR0449 – ST126 – P150
MSHR0462 – ST126 – P157
MSHR0491 – ST126 – Water tank

**Fig S2. Phylogenetic reconstruction in comparison to predicted recombination block distribution of the ST-125 and ST-126 strains.**

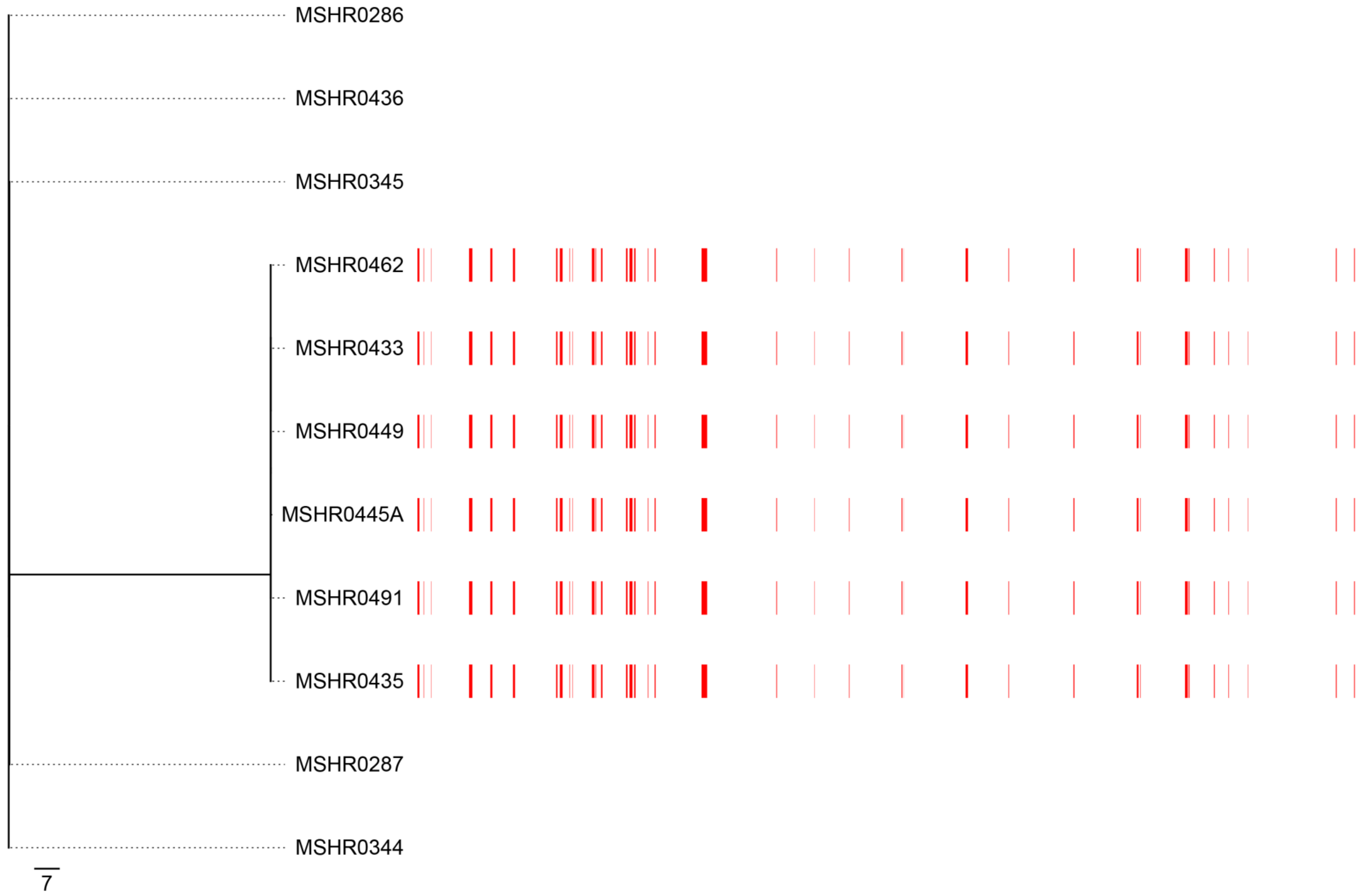
MSHR0286
MSHR0436
MSHR0345
MSHR0462
MSHR0433
MSHR0449
MSHR0445A
MSHR0491
MSHR0435
MSHR0287
MSHR0344
7

**Fig S3. Phylogenetic reconstruction in comparison to genome wide single-nucleotide polymorphism distribution of the ST-125 and ST-126 strains.**

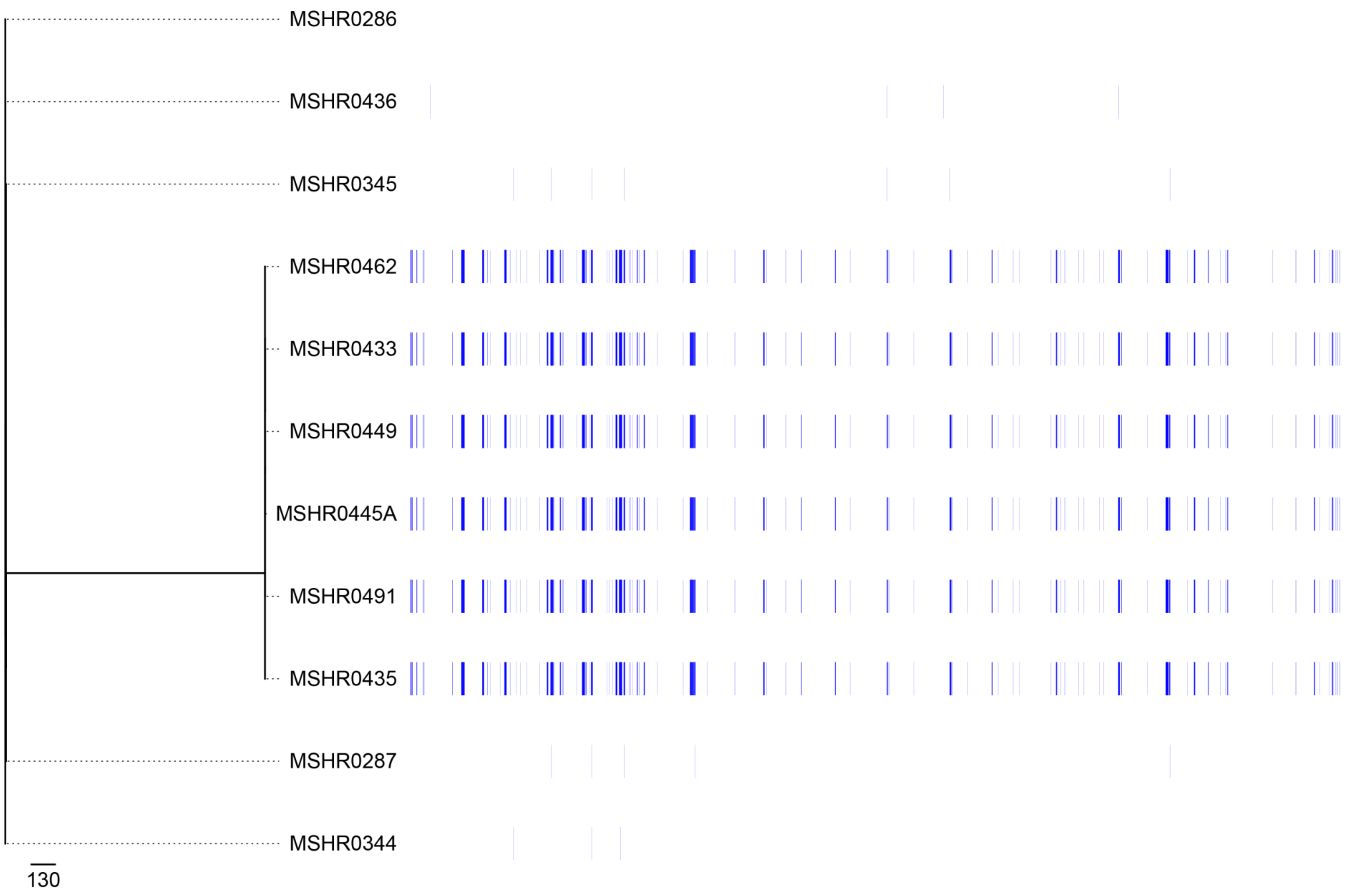
MSHR0286
MSHR0436
MSHR0345
MSHR0462
MSHR0433
MSHR0449
MSHR0445A
MSHR0491
MSHR0435
MSHR0287
MSHR0344
130

**Table S1: Summary of recombination prediction for ST-125 and ST-126 strains using Gubbins**

| Node | Total SNPs | SNPs inside recombination blocks | SNPs outside recombination blocks | Recombination Blocks | Bases in Recombination blocks | Recombination to mutation ratio | Bases in Clonal Frame |
|---|---|---|---|---|---|---|---|
| **MSHR0286** | 0 | 0 | 0 | 0 | 0 | 0 | 7356446 |
| **MSHR0287** | 2 | 0 | 2 | 0 | 0 | 0 | 7356446 |
| **MSHR0344** | 3 | 0 | 3 | 0 | 0 | 0 | 7356446 |
| **MSHR0345** | 4 | 0 | 4 | 0 | 0 | 0 | 7356446 |
| **MSHR0433** | 1 | 0 | 1 | 0 | 339312 | 0 | 7017098 |
| **MSHR0435** | 2 | 0 | 2 | 0 | 339312 | 0 | 7017098 |
| **MSHR0436** | 4 | 0 | 4 | 0 | 0 | 0 | 7356446 |
| **MSHR0445A** | 0 | 0 | 0 | 0 | 339312 | 0 | 7017098 |
| **MSHR0449** | 0 | 0 | 0 | 0 | 339312 | 0 | 7017098 |
| **MSHR0462** | 1 | 0 | 1 | 0 | 339312 | 0 | 7017098 |
| **MSHR0491** | 0 | 0 | 0 | 0 | 339312 | 0 | 7017098 |
| **Node5*** | 1343 | 1270 | 73 | 36 | 339312 | 17.4 | 7356446 |
| **Node3*** | 3 | 0 | 3 | 0 | 0 | 0 | 7356446 |

*Node 5= Separation between ST-125 and ST-126; Node 3= Node shared between MSHR0344 and MSHR0345. N.B. Nodes with no variation have been removed from this table. Abbreviations: SNP, single-nucleotide polymorphism.
